# Breast Cancer Screening Knowledge and Sentiments in Singaporean Women: Mixed Methods Study Using Topic Modeling, Sentiment Analysis, and Structured Questionnaire Data

**DOI:** 10.2196/78439

**Published:** 2026-03-10

**Authors:** Peh Joo Ho, Zi Lin Lim, Jenny Liu, Nur Khaliesah Mohamed Riza, Ying Jia Chew, Yi Ying Lim, Hui Ling Tan, Su-Ann Goh, Han Boon Oh, Chi Hui Chin, Sing Cheer Kwek, Zhi Peng Zhang, Desmond Luan Seng Ong, Swee Tian Quek, Sujith Wijerathne, Philip Tsau Choong Iau, Mikael Hartman, Jingmei Li

**Affiliations:** 1 Saw Swee Hock School of Public Health, National University of Singapore and National University Health System, Singapore, Singapore; 2 Department of Surgery, Yong Loo Lin School of Medicine, National University of Singapore and National University Health System, Singapore, Singapore; 3 Genome Institute of Singapore, Agency for Science, Technology and Research (A*STAR), #02-01, 60 Biopolis Street, Singapore, 138672, Singapore, 65 6808 8312; 4 Department of General Surgery, Ng Teng Fong General Hospital, Singapore, Singapore; 5 Department of Surgery, National University Hospital and National University Health System, Singapore, Singapore; 6 Department of General Surgery, Jurong Medical Centre, Singapore, Singapore; 7 Jurong Polyclinic, National University Polyclinics and National University Health System, Singapore, Singapore; 8 Bukit Batok Polyclinic, National University Polyclinics and National University Health System, Singapore, Singapore; 9 Choa Chu Kang Polyclinic, National University Polyclinics and National University Health System, Singapore, Singapore; 10 Department of Diagnostic Imaging, National University Hospital and National University Health System, Singapore, Singapore; 11 National Cancer Centre Singapore, Singapore Health Services (SingHealth), Singapore, Singapore

**Keywords:** mammography screening, breast cancer, Asian population, screening attitudes, breast cancer awareness, precision health, proactive health

## Abstract

**Background:**

Mammography screening uptake in Singapore remains below 40% despite campaigns and subsidies. Natural language processing (NLP) can extract nuanced attitudes from free text that fixed response options miss, revealing latent factors influencing breast cancer (BC) screening behavior.

**Objective:**

This study characterized women’s attitudes toward mammography using mixed methods data, examined associations between BC awareness and screening willingness, and identified barriers and facilitators through NLP of free-text responses.

**Methods:**

We conducted a cross-sectional study within the *Breast Screening Tailored for Her* multicenter cohort in Singapore (October 2021-December 2023). In total, 4169 women aged 35‐59 years (median 48, IQR 43‐54) were recruited via convenience sampling (3 hospitals and 2 polyclinics). Participants completed online structured questionnaires on demographics and screening history, then a BC education quiz with feedback. Participants answering >80% correctly were classified as “BC-aware.” Posteducation, participants reported screening willingness (motivated or neutral) with optional free-text explanations. Logistic regression models (adjusted for study site, age, ethnicity, marital status, housing, and education) examined the associations with willingness. For 3819 English-language respondents, biterm topic modeling identified themes and sentiment analysis quantified emotional tone. Statistical significance: *α*=.05.

**Results:**

Overall, 79% (3287/4169) were BC-aware, and 94% (3908/4169) reported increased motivation posteducation. BC-aware women had higher screening motivation than BC-unaware women (adjusted odds ratio [aOR] 2.88, 95% CI 2.19‐3.80; *P*<.001). Motivation was higher among those with larger public housing (OR 1.81, 95% CI 1.30‐2.50; *P*<.001) and private housing vs 1‐3 room units (OR 2.69, 95% CI 1.75‐4.13; *P*<.001), married vs not separated, divorced, or widowed (OR 2.38 [inverse of 0.42], 95% CI 1.75‐3.13; *P*<.001), and prior screening attendance (OR 3.49, 95% CI 2.71‐4.50; *P*<.001). Women who disagreed that mammography was expensive had higher motivation (aOR 1.94, 95% CI 1.50‐2.50; *P*<.001). Among 3819 English respondents, 94% (3579/3819) were motivated and 6% (240/3819) neutral. Free-text responses came from 34% (1220/3579) of motivated and 64% (153/240) of neutral participants. Biterm topic modeling revealed motivated participants emphasized early detection benefits, health awareness, BC risk, and logistics; neutral participants focused on mammography pain experiences and cost barriers. Mean sentiment was 0.207 (range: −1.00 to 1.65), with motivated participants displaying more positive sentiments than neutral participants (linear regression, *P*<.001). Identical words carried different emotional tones across subgroups: “health” had positive sentiment among motivated participants (mean difference, Welch *t* tests *P*<.05) but negative sentiment among neutral participants. Word frequency analysis showed motivated participants used positive-sentiment words (“better,” “cure,” and “prevention”). Neutral participants emphasized negative words (“painful” and “uncomfortable”).

**Conclusions:**

Integrating quantitative surveys with NLP revealed that the same screening concepts are emotionally framed differently by motivated vs neutral women, a finding missed by knowledge- or intent-focused approaches alone. In practice, these findings support the need for emotionally tailored BC education and prevention strategies.

## Introduction

Mammography screening has been widely regarded as the most effective method for achieving early detection of breast cancer (BC), leading to improved survival [1-3]. Despite a decade of annual campaigns and various subsidies, the mammography screening rate remains suboptimal, even among women who know about mammogram services in Singapore [4,5].

A review by Momenimovahed et al [6] found that barriers to mammography screening in Asia include factors such as financial limitations, lack of social support, fatalistic tendencies, fear of pain and embarrassment, and religion. Barriers to mammography screening specific to Singapore have also been widely examined in multiple studies, and the common obstacles reflected are the lack of time, fatalism, misconceptions about mammography screening or BC, reluctance to spend on screening, and religious reasons [7-12]. In a review by Rajendram et al [4], the perceived costs of screening played the biggest role in hindering screening participation among Singaporean women.

According to the principle of attitude consistency, a person’s attitudes tend to influence and shape their actions and behavior [13]. Common techniques used to understand people’s opinions and attitudes toward screening include quantitative tools, such as the Likert scale, or qualitative tools, such as focus groups or in-depth interviews. However, these tools are limited by a few factors. Self-reported measurements using the Likert scale often present participants with closed-ended scenarios. The finite number of response options can limit respondents’ ability to express nuanced opinions, and responses can be prone to central tendency bias [14,15]. On the other hand, while focus groups and in-depth interviews offer valuable qualitative data, providing context and information on nuances of responses, they are limited by factors such as response bias [16], researcher bias, and time-intensive analysis processes [17].

In recent years, natural language processing (NLP) has emerged as a novel tool in health behavior and health services research, offering unique advantages in understanding individuals’ attitudes and perceptions [18,19]. By leveraging machine learning algorithms, NLP can sift through vast amounts of textual information to extract nuanced insights [20,21]. This approach not only enables researchers to capture the rich complexity of human language but also serves as an avenue to reveal implicit sentiments and identify emerging themes. Subsequently, the use of NLP can offer a more comprehensive understanding of the factors influencing the uptake of health behaviors, such as screening.

Currently, studies involving the use of NLP tools in understanding health-seeking behavior largely revolve around social media posts [22]. A systematic review by Döbrössy et al [23] demonstrated the value of social media as a platform to spread BC awareness and the value of NLP tools in identifying common areas of concern of the lay public about BC. NLP’s ability to analyze free text provides valuable insights into the psychological and social factors influencing health behaviors, potentially contributing to more effective public health interventions and communication strategies. This study leverages structured questionnaire data supplemented by NLP tools to analyze free-text responses, aiming to (1) characterize women’s attitudes toward mammography, (2) examine the relationship between BC awareness and screening willingness, and (3) identify barriers and facilitators for the willingness to screen.

## Methods

### Study Design

We conducted a cross-sectional, observational, mixed methods study combining quantitative surveys and qualitative free-text analysis to examine women’s attitudes and willingness to attend routine mammography screening. This study was reported following the American Psychological Association Journal Article Reporting Standards for mixed-methods, quantitative, and qualitative research [24].

### Setting

The Breast Screening Tailored for Her (BREATHE) study is a multicenter prospective cohort study in Singapore [25,26]. Participants were recruited between October 2021 and December 2023 from 3 restructured hospitals—Ng Teng Fong General Hospital, National University Hospital, and Alexandra Hospital—2 polyclinics—Bukit Batok Polyclinic and Choa Chu Kang Polyclinic—and Jurong Medical Center [25]. Briefly, potential participants were identified through advertisements (eg, posters, flyers, and blog recruitment pages inviting women to register interest) and an active approach at the clinical sites (ie, study staff approached women in waiting areas of the participating hospitals and polyclinics). Recruitment was based on convenience (ie, convenience sampling) and recruitment feasibility across participating clinical sites rather than a formal a priori power calculation.

Eligible participants were female, aged between 35 and 59 years, and Singapore citizens or permanent residents at the point of enrollment. Participants were excluded if they had a histologically confirmed diagnosis of any cancer, were cognitively impaired, or were pregnant during recruitment. Consistent with the subsidized Screen for Life BC screening criteria in Singapore, women with benign breast disease were eligible for the study, while those with breast implants were excluded. After providing informed consent, participants completed an online structured first-visit questionnaire, which included factors linked to BC and related conditions, such as demographic, lifestyle, and reproductive characteristics, prior treatments, and other environmental exposures (Multimedia Appendix 1). They then undertook a brief, self-administered education session online covering BC knowledge and the importance of regular breast self-examination and screening (Multimedia Appendix 2). Participants answered the online education questionnaire in either English, Chinese, or Malay.

A flowchart illustrating the selection of participants from those enrolled in the BREATHE study is shown in Figure S1 in Multimedia Appendix 3. Between October 2021 and December 2023, 4592 participants were enrolled in the study (corresponding to a participation rate of 83% among the 5536 women approached or expressing interest). Participants were excluded if they withdrew from the study (n=74), were diagnosed with BC within 6 months of enrollment (n=17), had unknown past mammography (n=38), or had incoherent BC screening and mammography screening attendance based on the lifestyle questionnaire and education session (n=294). The analytical cohort comprised 4169 participants. The free-text analysis included participants who answered the English questionnaire (n=3819).

### Demographics and BC Risk Factors

Baseline information on demographics and BC risk factors was obtained from the first-visit questionnaire. The variables included ethnicity, medical history, previous benign lump or gynecological surgery (yes, no, or missing), family history of breast and ovarian cancer (yes, no, or missing), reproductive factors, and previous breast examination and screening habits, among others.

Sociodemographic factors were derived from the same questionnaire, where individual factors were further categorized for ease of analysis (Table S1 in Multimedia Appendix 4). Housing (public: 1‐3 room flat, public: >3-room flat [4-, 5-, or executive-type], or private), highest qualification achieved (no formal or primary, secondary, postsecondary [nontertiary], professional diploma, or tertiary), and marital status (married, never married, widowed, or separated or divorced) were used as proxies for economic, education, and social support status, respectively.

### BC Education

Participants’ existing screening habits and views about BC were assessed during an online education session (Multimedia Appendix 2). Various statements were used to assess BC awareness and provide education (questions 7‐13, 16, and 18). These statements were presented to participants to indicate their agreement (agree or disagree). The correct answer and an accompanying explanation were given after every response. Participants were characterized as aware of BC risk (ie, BC-aware) before study enrollment if they answered >80% of the questions correctly.

Following BC education, participants were asked about their willingness to attend regular screening (question 20: “After knowing the above, would you be more willing to attend regular screening?” yes or no) and were categorized as “motivated” or “neutral,” respectively. Participants were also asked to explain their choice (question 21: “Referring to your answer in the last question, please provide a reason.” free text, optional).

Statements unrelated to BC or BC screening knowledge were considered as BC perceptions for analysis (ie, perceived importance of BC screening, perceived risk of BC, fatalistic attitudes, and finding mammography embarrassing, expensive, inconvenient, or painful).

### Data Analysis

Descriptive analyses were conducted to characterize demographic factors and BC screening attitudes. The associations between willingness to attend more regular screening and the various characteristics were studied using the chi-square and Kruskal-Wallis tests for categorical and continuous variables, respectively.

The study’s primary outcome was self-reported willingness to screen, measured immediately after an education intervention. The association between the various factors and participants’ willingness to attend regular screening was studied using logistic regression models. In addition to univariate analysis, the models were adjusted for study site and participant characteristics (age category, ethnicity, marital status, housing type, and highest academic status). To select the best predictive model, stepwise forward selection was used (ie, the lowest Bayesian information criterion). Odds ratios (ORs) and 95% CIs are presented.

### Free-Text Data Preprocessing

The sentiment of words may not be well captured through translation. We thus retained only the reasons provided in English in the sentiment analysis. Of the 4169 participants, 3819 (92%) completed the English version of the education session. Participants (n=350) who completed the Chinese or Malay version of the education session were excluded from text analysis. Free-text analysis was done to assess participants’ sentiments toward regular screening. Each participant’s response was tokenized (R package *tidytext;* version 0.4.1, Julia Silge and David Robinson) [27], and spelling errors were corrected (R package *hunspell;* version 3.0.4, Jeroen Ooms). Additional spelling errors and corrections were added to the list generated. The full spelling errors and corrections can be found in Table S2 in Multimedia Appendix 4. The participants were categorized by BC awareness (BC-aware vs BC-unaware) and their willingness to attend regular screening in the future posteducation session (motivated vs neutral; Figure S1 in Multimedia Appendix 3).

### Biterm Topic Modeling

Biterm topic modeling (R package *BTM*; version 0.3.7, Jan Wijffels) was used to identify the top topics mentioned by the participants [28]. Although there are other topic modeling methods, such as latent Dirichlet allocation, latent semantic analysis, nonnegative matrix factorization, and transformer-based approaches such as BERTopic, biterm topic modeling was chosen due to the short length of the text inputs (1-3 sentences), its computational efficiency, and the interpretability of the resulting topics [29,30]. As the choice of the number of topics in the topic model will impact the effect of the model, we compared the results of the models with 2-5 topics (ie, parameter k). Expecting a small number of topics within each participant’s response, we set α=.01 and *ß*=.01. The clustering of words into topics depends on the initialization step and may change with different iterations. We attempted 6 iterations for each k, using set.seed() and permutations of 123 (ie, 123, 132, 213, 231, 312, and 321). The choice of the number of topics (k) was based on the stability of the topics produced across iterations, and the topic for each set of words was derived by reviewing the comments for relevant words and discussing them with team members. Biterm topic modeling looks at pairs of words and the probability of either of the words being in a particular topic. Topics emerged from the data rather than being predetermined, with the biterm topic modeling algorithmically identifying word co-occurrence patterns without a priori coding categories. This requires word stemming (R package *hunspell*; version 3.0.4) to prevent words with the same meaning from being tokenized into different tokens (eg, the words “prevented” and “prevents” have the same stem word “prevent,” but not “prevention”) and removing stop words from the text corpus. The list of stop words was derived from “stop_words” in tidytext and was further edited to suit this study. The full list of stem words and stop words is provided in Tables S3 and S4 in Multimedia Appendix 4, respectively.

### Sentiment Analysis

In our study, “sentiment” refers to the emotional tone of participants’ free-text responses as classified by NLP algorithms and used strictly as a quantitative analytical construct rather than a synonym for perception, attitude, or feeling. Unlike biterm modeling, which uses all pairs of words, sentiment analysis analyzes sentences or single words. Here, sentiment analysis was done using the R package *sentimentr;* version 2.9.0, Tyler W Rinker), as it accounts for valence shifters to determine the sentiment at the sentence level, while estimating the sentiment score by referencing the Jockers sentiment dictionary. Because these free-text responses were brief (1-3 sentences) and expressed lay perspectives and emotions about mammography screening rather than technical medical terminology, the use of a general-purpose sentiment lexicon with minor modifications was appropriate and ensured consistent capture of participants’ attitudes across the cohort. The package scores sentiments on a scale where 0 is considered neutral, negative numbers indicate the presence of negative sentiments, and positive numbers indicate the presence of positive sentiments. Sentiment rating of certain words was further modified to suit the context of the study (Table S5 in Multimedia Appendix 4). Free-text responses to question 21 in Multimedia Appendix 2 were first analyzed by sentence and then averaged across sentences for each participant. Linear regression was used to determine significant differences in sentiment ratings across subgroups.

Word clouds (R package *ggplot2;* version 3.4.4, Hadley Wickham) were used to tabulate the top sentiment words of each subgroup. The larger the word in the visual, the more common the word was in the subgroup. To further explore patterns, common sentiment words, and the average sentiment associated were compared across subgroups using the Welch 2-sample *t* test. Unique sentiment words were also extracted. Example quotes of common and unique words were extracted randomly to provide better context.

### Interpretive Limitations

The research team comprised epidemiologists, public health researchers, and data scientists with expertise in cancer screening behavior and computational text analysis. The use of algorithmic biterm topic modeling and sentiment analysis minimizes researcher interpretation bias. Researchers’ perspectives influenced only the selection of analytical methods and the labeling of algorithmically identified topic clusters. Rather than sampling to thematic saturation as in traditional qualitative research, we analyzed all available free-text responses. Data collection concluded at the end of the study recruitment period (December 2023) rather than when thematic saturation was achieved. The stability of topics identified across 6 biterm topic modeling iterations with different random seeds provides evidence of thematic robustness within our dataset. The relatively brief nature of responses (1‐3 sentences) and focus on specific questions means our analysis captures targeted attitudes toward screening rather than comprehensive life narratives, which is appropriate for our research objectives.

### Ethical Considerations

This study involved human participants and complied with all relevant institutional and national research ethics guidelines. Ethics approval was obtained from the National Healthcare Group Domain-Specific Review Board, Singapore (reference no 2020/01327; approval date: June 7, 2021). Written informed consent was obtained from participants by trained study coordinators in the participant’s preferred language (English, Chinese, or Malay). The informed consent process included permission to use the study data for secondary analyses relevant to BC screening research; therefore, no additional consent was required for the current analysis. Participant privacy and confidentiality were safeguarded throughout the study. All research data were deidentified before analysis, stored on secure servers with restricted access, and handled in accordance with institutional data protection policies to ensure anonymity. No individual participants can be identified in any image in the paper or multimedia appendices.

## Results

### Participant Characteristics

Table 1 shows the descriptive statistics of participant characteristics. Of the 4169 participants included, 3908 (94%) indicated that they were more motivated to attend regular screening posteducation sessions. The median age of the participants was 48 years (IQR 43-54), and the majority (77%, n=3208) of the participants were of Chinese ethnicity. Overall, 79% (n=3287) of the participants were BC-aware, and 22% (n=938) and 41% (n=1690) attended BC screening once every year or once every 2 years, respectively. Seventy-three percent (n=3056) had done at least 1 mammogram in the past. While 44% (n=1845) perceived themselves as low risk and 47% (n=1944) as average risk, 96% (n=4023) of the participants agreed that BC screening is important. Twelve percent (n=485) of the participants reported a history of benign breast disease.

**Table 1.** Characteristics of the BREAst Screening Tailored for HEr (BREATHE) study population, by their willingness to attend screening. Participants were recruited between 2021 and 2023 in Singapore’s health care institutes. Two-sided *P* values for categorical variables are based on the chi-square test, and *P* values for continuous variables are based on the Kruskal-Wallis test. Counts and column percentages are presented, unless otherwise stated.

Characteristic	Total, n=4169, n (%)	Neutral, n=261, n (%)	Motivated, n=3908, n (%)	*P* value
BC^a^ awareness				<.001
BC-unaware	882 (21)	104 (40)	778 (20)	
BC-aware	3287 (79)	157 (60)	3130 (80)	
Age, median (IQR)	48 (43-53)	49 (44-53)	48 (42-53)	.45
Age category (years)				.33
35-39	531 (13)	35 (13)	496 (13)	
40-49	1846 (44)	104 (40)	1742 (45)	
50-59	1792 (43)	122 (47)	1670 (43)	
Participant characteristic				
Ethnicity				.96
Chinese	3208 (77)	204 (78)	3004 (77)	
Malay	465 (11)	28 (11)	437 (11)	
Indian	279 (7)	17 (7)	262 (7)	
Other	217 (5)	12 (5)	205 (5)	
Marital status				<.001
Married	3186 (76)	164 (63)	3022 (77)	
Separated, divorced, or widowed	349 (8)	25 (10)	324 (8)	
Never married	634 (15)	72 (28)	562 (14)	
Housing				<.001
Public 1-3 room	490 (12)	52 (20)	438 (11)	
Public >3 room	2757 (66)	170 (65)	2587 (66)	
Private	922 (22)	39 (15)	883 (23)	
Highest qualification attained				.06
No formal or primary	235 (6)	23 (9)	212 (5)	
Secondary	787 (19)	46 (18)	741 (19)	
Postsecondary	1240 (30)	66 (25)	1174 (30)	
Tertiary	1907 (46)	126 (48)	1781 (46)	
Self-reported breast cancer history, existing behavior, and perceptions				
Family history of BC				.94
No	3756 (90)	236 (90)	3520 (90)	
Yes	413 (10)	25 (10)	388 (10)	
Benign breast disease				.25
No	3673 (88)	235 (90)	3438 (88)	
Yes	485 (12)	24 (9)	461 (12)	
Unknown	11 (0)	2 (1)	9 (0)	
BCS^b^ attendance				<.001
No	1113 (27)	140 (54)	973 (25)	
Yes	3056 (73)	121 (46)	2935 (75)	
Mammography attendance				<.001
No	1113 (27)	140 (54)	973 (25)	
Yes	3056 (73)	121 (46)	2935 (75)	
Previous BCS type				<.001
Mammography	3056 (73)	121 (46)	2935 (75)	
Ultrasound only	94 (2)	7 (3)	87 (2)	
Neither	1019 (24)	133 (51)	886 (23)	
Current BCS behavior				<.001
Once a year	938 (22)	11 (4)	927 (24)	
Once every 2 years	1690 (41)	32 (12)	1658 (42)	
Do not intend to attend in the future	99 (2)	43 (16)	56 (1)	
Other	320 (8)	35 (13)	285 (7)	
Not in the above categories and aged 35-39 years	506 (12)	35 (13)	471 (12)	
Unknown	616 (15)	105 (40)	511 (13)	
Perceived importance of BCS				<.001
Agree	4023 (96)	198 (76)	3825 (98)	
Neutral	136 (3)	57 (22)	79 (2)	
Disagree	10 (0)	6 (2)	4 (0)	
Perceived risk of BC				.03
Low	1845 (44)	133 (51)	1712 (44)	
Average	1944 (47)	101 (39)	1843 (47)	
High	380 (9)	27 (10)	353 (9)	
Fatalism toward developing BC				.85
Low	2002 (48)	121 (46)	1881 (48)	
Average	1179 (28)	77 (30)	1102 (28)	
High	988 (24)	63 (24)	925 (24)	
Fatalism toward dying from BC				.22
Low	2456 (59)	153 (59)	2303 (59)	
Average	1023 (25)	73 (28)	950 (24)	
High	690 (17)	35 (13)	655 (17)	
Finds mammography: embarrassing				<.001
Agree	586 (14)	74 (28)	512 (13)	
Disagree	3583 (86)	187 (72)	3396 (87)	
Expensive				<.001
Agree	1663 (40)	144 (55)	1519 (39)	
Disagree	2506 (60)	117 (45)	2389 (61)	
Inconvenient				<.001
Agree	866 (21)	105 (40)	761 (19)	
Disagree	2779 (67)	120 (46)	2659 (68)	
Not applicable	524 (13)	36 (14)	488 (12)	
Painful				<.001
Agree	1027 (25)	134 (51)	893 (23)	
Disagree	2618 (63)	91 (35)	2527 (65)	
Not applicable	524 (13)	36 (14)	488 (12)	

a BC: breast cancer.

b BCS: breast cancer screening.

### BC Awareness Is Significantly Associated With Posteducation Screening Motivation

In univariate logistic models, more motivated participants were more likely to be BC aware (OR 2.67, 95% CI_aware vs unaware_ 2.05-3.46; *P*<.001), residing in >3 rooms HDB (OR_>3 rooms HDB vs 1-3 rooms HDB_1.81, 95% CI 1.30-2.50; *P*<.001) or private housing (OR _private vs 1-3 rooms HDB_ 2.69, 95% CI 1.75-4.13; *P*<.001), currently married (OR_separated/divorced/widowed vs currently married_ 0.42, 95% CI 0.32-0.57; *P*<.001), having attended BC screening before (OR_yes vs no_ 3.49, 95% CI 2.71-4.50; *P*<.001), being currently regular screeners, and being less likely to find the procedure embarrassing, expensive, inconvenient, or painful (Table 2). The complete results table of univariate analysis and the results adjusting for participants’ characteristics is provided in Table S6 in Multimedia Appendix 4.

**Table 2.** Associations between patient characteristics and willingness to attend regular screening posteducation. Stepwise forward selection was used to select the best predictive model (ie, the lowest Bayesian information criterion [BIC]).

Characteristic	Univariate		Best prediction model by BIC	
	OR (95% CI)	*P* value	OR (95% CI)	*P* value
BC^a^ awareness		<.001		<.001
BC-unaware	1.00 (referent)		1.00 (referent)	
BC-aware	2.67 (2.05-3.46)		1.90 (1.41-2.56)	
Age category (years)				
35-39	1.00 (referent)	—^b^	—	—
40-49	1.18 (0.80-1.76)	.41	—	—
50-59	0.97 (0.65-1.43)	.86	—	—
Ethnicity				
Chinese	1.00 (referent)	—	—	—
Malay	1.06 (0.71-1.59)	.78	—	—
Indian	1.05 (0.63-1.74)	.86	—	—
Other	1.16 (0.64-2.11)	.63	—	—
Marital status				
Married	1.00 (referent)	—	1.00 (referent)	—
Never married	0.70 (0.45-1.09)	.11	0.80 (0.49-1.31)	.38
Separated, divorced, or widowed	0.42 (0.32-0.57)	<.001	0.46 (0.33-0.64)	<.001
Family history of breast cancer				
No	1.00 (referent)	—	—	—
Yes	1.04 (0.68-1.59)	.86	—	—
BCS^c^ attendance				
No	1.00 (referent)	—	—	—
Yes	3.49 (2.71-4.50)	<.001	—	—
Previous BCS type				
Mammography	1.00 (referent)	—	—	—
Ultrasound only	0.51 (0.23-1.13)	.10	—	—
Neither	0.27 (0.21-0.36)	<.001	—	—
Current BCS behavior				
Once a year	1.00 (referent)	—	1.00 (referent)	—
Once every 2 years	0.61 (0.31-1.23)	.17	0.65 (0.33-1.30)	.23
Do not intend to attend in the future	0.02 (0.01-0.03)	<.001	0.03 (0.01-0.06)	<.001
Other	0.10 (0.05-0.19)	<.001	0.13 (0.07-0.27)	<.001
Not in above categories and aged 35-39 years	0.16 (0.08-0.32)	<.001	0.17 (0.03-0.87)	.03
Unknown	0.06 (0.03-0.11)	<.001	0.10 (0.05-0.20)	<.001
Perceived importance of BCS				
Agree	1.00 (referent)	—	1.00 (referent)	—
Neutral	0.07 (0.05-0.10)	<.001	0.21 (0.14-0.32)	<.001
Disagree	0.03 (0.01-0.12)	<.001	0.11 (0.02-0.50)	.004
Finds mammography painful				
Agree	1.00 (referent)	—	1.00 (referent)	—
Disagree	4.17 (3.16-5.50)	<.001	2.08 (1.52-2.85)	<.001
Not applicable	2.03 (1.39-2.99)	<.001	1.95 (0.43-8.80)	.39

a BC: breast cancer.

b Not applicable.

c BCS: breast cancer screening.

After adjustment for study site and participant characteristics (age, ethnicity, marital status, housing type, highest academic status, and family history of BC), BC awareness remained significant (OR_aware vs unaware_ 2.88, 95% CI 2.19-3.80; *P*<.001) (Table S6 in Multimedia Appendix 4). The best combination of factors (according to the lowest Bayesian information criterion) that predicts screening motivation was BC awareness, marital status, current BC screening behavior, perceived importance of BC screening, and pain during mammography.

### Demographic Differences in Participants Using the English and the Non-English Questionnaire

Participants who used the English version of the questionnaire (n=3819) were more likely to be BC aware, younger, and living in larger housing, and had higher educational attainment, as compared to participants who used the non-English questionnaire (n=350) (Table S7 in Multimedia Appendix 4 ). A total of 350 participants answered the Chinese or Malay versions of the questionnaire, of which 31 (9%) answered the free-text question on reasons for screening.

### Differences in Characteristics Between Participants Who Provided and Did Not Provide Free-Text Explanation

Participants who provided and did not provide an explanation were not statistically different in age, ethnicity, housing type, and family history of BC (Table S8 in Multimedia Appendix 4). Participants who provided explanations were more likely to have never been married (*P*=.04) and to have higher educational attainment (*P*<.001). These participants differed from those who did not provide free-text responses regarding their current BC screening behavior (*P*=.01), perceived importance of screening (*P*=.002), perceived risk of BC (*P*=.01), and reporting that mammography was painful (*P*=.004) (Table S8 in Multimedia Appendix 4).

### Biterm Topic Modeling Reveals Key Themes by Motivation Level

Of the 3819 participants who answered in English, 94% (n=3579) were motivated, and 6% (n=240) remained neutral about regular screening after the breast education session. Among the motivated participants, 1220 (34%) provided additional comments, compared to 153 (64%) among the neutral participants. Tables S9 and S10 in Multimedia Appendix 4 provide the sets of words derived from biterm topic modeling. The top and bottom 20 words are listed.

In the motivated participants, we selected 4 topics as repeats in top words occurred at 5 topics (Table S9 in Multimedia Appendix 4). “Early” and “health” were consistent across all 6 iterations, with “age” and “appointment” occurring 4 times, “better” twice, and “risk” and “painful” once. It was observed that “age” and “risk” belonged to the same set, while “appointment,” “better,” and “painful” were used commonly together. Using the first iteration (Table 3), topics observed were (1) benefits of early detection (with words “early,” “detection,” “better,” “cure,” and “prevention”), (2) health awareness (“health,” “regular,” “know,” “check,” and “important”), (3) BC risk (“age,” “risk,” “40,” “2,” and “safe”), where 40 refers to the age of 40 years and 2 refers to screening once every 2 years, (4) logistics and mammography experience (“appointment,” “regular,” “routine,” “schedule,” and “painful”).

In the neutral participants, we selected 2 topics as repeats in the top words that occurred at 3 topics (Table S10 in Multimedia Appendix 4). No word occurred in all iterations. “Pain” and “painful” were from the same set of words; “time,” “regular,” “health,” and “examination” were commonly from the same set of words. Using the first iteration (Table 3), unlike in the motivated participants, there was a distinction between (1) mammography experience with a focus on pain during a mammogram and (2) logistics, with a mention of “expensive” that was not observed in the top words from motivated participants. This is in line with our finding that participants who disagreed that mammography was expensive were more likely (OR_disagree vs agree_ 1.94, 95% CI 1.50-2.50) to be willing to attend the screening posteducation session (Table S6 in Multimedia Appendix 4).

**Table 3.** Topics surfaced using biterm topic modeling on the free-text response following the question, “After knowing the above, would you be more willing to attend regular screening?” by willingness to screen: motivated and neutral. As different iterations with the same number of clusters produce variations in results, the first iteration is presented. Topic distribution (θ) provides the proportion of each topic in the entire corpus (dataset of additional comments). “k” refers to the number of clusters and was selected based on the occurrence of repeated topics within each iteration.

Cluster	Value (θ)	Top word	Subsequent 19 words	Topic
Motivated participants (n=1205)				
1	0.550	Early	Detection, better, cure, detect, prevention, chance, treatment, life, save, regular, survival, important, increase, live, recovery, believe, stage, always	Benefits of early detection
2	0.248	Health	Early, regular, good, know, check, important, detection, always, time, body, need, prevent, condition, family, age, risk, better, detect	Health awareness
3	0.103	Age	Risk, regular, 40, 2, safe, know, need, check, reduce, women, test, health, importance, 50, diagnose, healthy, aware, yearly	Breast cancer risk
4	0.099	Appointment	Health, better, regular, routine, follow, schedule, painful, women, easy, important, preventive, experience, hard, purpose, issue, patient, question, yearly	Logistics and mammogram experience
Neutral participants (n=145)				
1	0.569	Pain	Time, 2, once, cost, follow, risk, 1, booking, know, change, comfortable, frequent, abnormality, body, chance, depend, factor, increasing	Mammogram experience
2	0.431	Regular	Health, health care, painful, need, expensive, 40, attend, examination, women, schedule, time, change, decision, subsidize, appointment, ease, good, impact	Logistics

### Subgrouping by BC Awareness Yields Similar Results to Motivation-Based Grouping

We repeated the analysis comparing BC-aware vs BC-unaware participants. Eighty-four percent (n=1010) of motivated women and 66% (n=95) of neutral women were BC-aware. When participants were grouped by BC awareness (n_BC-aware_=1105 and n_BC-unaware_=243), 2 topics were identified for BC-aware (ie, benefits of early detection and health awareness), similar to the motivated group (Table S11 in Multimedia Appendix 4). In BC-unaware participants, the main topic identified was the benefits of early detection (Table S12 in Multimedia Appendix 4). A second topic was not identified, as the word sets varied across the 6 iterations, likely reflecting the limited thematic diversity of responses. Conclusions about additional potential topics should be interpreted carefully.

### More Motivated Participants Displayed More Positive Sentiments Toward Regular Screening

**Figure 1.**
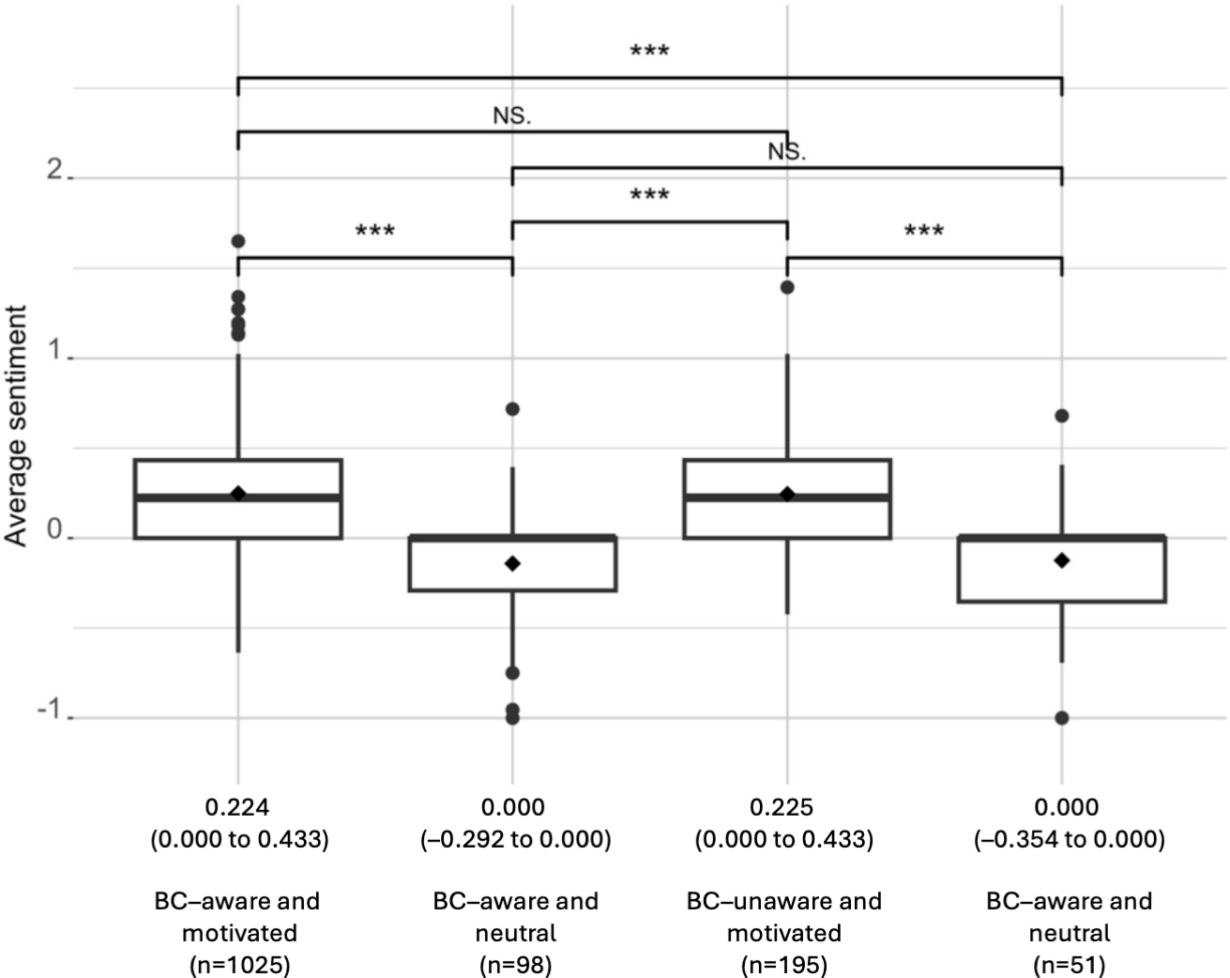
Comparison of average sentiments across groups according to breast cancer (BC) awareness and willingness to screen (♦=mean). Median sentiment and IQR values of each subgroup are displayed at the bottom. Differences in sentiments were tested using the Wilcoxon test (***P<.001, **P<.01, and *P<.05). NS: not significant.

The minimum sentiment rating obtained in the analysis was −1.00, and the maximum rating was 1.65. The median and mean of the sentiment scores were 0.177 (IQR 0.000-0.202) and 0.202 (SD 0.327), respectively, indicating that positive sentiments were more prevalent than negative ones among the participants. The mean sentiment values and 95% CIs of each group are displayed in Figure 1. Motivated participants had significantly more positive sentiments toward regular screening than neutral participants, regardless of existing BC awareness.

### Word Frequencies are Indicative of Subgroup Sentiments

Words, expressed by at least 5% of each subgroup, were summarized and tabulated (Table 4). Motivated participants, both BC-aware and BC-unaware, displayed higher frequencies of words with positive sentiments (eg, “better,” “cure,” and “prevention”). Neutral participants who were BC-unaware displayed higher frequencies of negative sentiment words (eg, “painful,” “pain,” and “uncomfortable”), and only “painful” had over 5% frequency in participants who were BC-aware (Table 4).

**Table 4.** Top words mentioned in the free-text responses following the question, “After knowing the above, would you be more willing to attend regular screening?” by breast cancer awareness (BC-aware or BC-unaware) and willingness to attend screening (motivated or neutral), were concluded using biterm topic modeling. Words were mentioned by at least 5% of participants in each respective subgroup.

Subgroups	Positive words	Neutral words	Negative words
BC-aware^a^ and motivated (n=1050)	Cure (n=74); better (n=136); important (n=80); detection (n=223); prevention (n=107); detect (n=68)	Early (n=362); health (n=101); regular (n=62)	—^b^
BC-aware and neutral (n=99)	—	Time (n=8); regular (n=8); health (n=6); cost (n=5)	Painful (n=15)
BC-unaware and motivated (n=206)	Better (n=24); important (n=19); detection (n=24); detect (n=22); prevention (n=17); prevent (n=13)	Early (n=48); health (n=20); regular (n=15); know (n=12)	—
BC-unaware and neutral (n=54)	Working (n=2); depends (n=2)	Time (n=6); necessary (n=3); regular (n=3); examination (n=3); cost (n=2); need (n=2); know (n=2)	Painful (n=8); troublesome (n=2); risk (n=2); pain (n=2); uncomfortable (n=2)

a BC: breast cancer.

b Not available.

### Comparison of Words Across Subgroups

To gain deeper insights into the context of words and the subtleties surrounding future screening willingness, comments on the common and unique words from each subgroup were extracted. Here, we considered words that occurred in at least 5% of the subgroup.

Table S13 in Multimedia Appendix 4 provides the common words (n=12), along with the average sentiment of comments involving the words and some example quotes. Four words (“health,” “important,” “know,” and “regular”) had different sentence sentiments when used by the different subgroups. “Health” was a common word across 3 subgroups (BC-aware and motivated, BC-aware and neutral, and BC-unaware and motivated). When used by a participant in the BC-aware and motivated group, the sentence had a positive sentiment (eg, “health check is important to take care of one’s health,” “I want to stay healthy to see my girl grow up”). However, participants in the BC-aware and neutral group used it in sentences that had a negative sentiment (“I find it inconvenient to go for health screening,” “the health care system does not allow women younger than 40 years to get subsidized breast screening”). Participants in the BC-unaware and motivated group were not significantly different from the BC-aware and motivated group (eg, “for my own health” and “it’s important to keep track of one’s health”).

“Know” was used by BC-unaware participants, among whom the motivated participants used “know” in positive sentiment sentences (eg, “better to know if there is a sign of BC and get treatment” and “I thought that screening is expensive and did not know that there are subsidies”). This highlighted the utility of the education session in increasing BC awareness. However, participants who remained neutral used “know” to express negative sentiments (eg, “knowing the above does not affect my regular screening” and “better not to know”), which shows the limitations of the education exercise.

“Regular,” a common word across all 4 subgroups, was used positively by motivated participants, for example, “important to do regular check-ups” by the BC-aware participants and “regular breast screening is important to detect any abnormality” by BC-unaware participants. “Regular” was used in negative sentences by the neutral participants, for example, “do not like too regular screening” by BC-aware participants and “I will be more open to regular screenings once I am 40” by BC-unaware participants.

Table 5 provides the words (n=4; “cure,” “examination,” “necessary,” and “prevent”) unique to the subgroup. While the words “examination” and “necessary” were mentioned by 3 participants (BC-unaware and neutral), the word “cure” was mentioned 74 times by BC-aware and motivated participants. The word “prevent” was mentioned 13 times by BC-unaware and motivated participants. It can be concerning that after the education session, participants used “prevent” regarding the occurrence of cancer (eg, “to prevent myself from any cancer”).

**Table 5.** Use of unique sentiment words in context according to breast cancer awareness (BC-aware or BC-unaware) and willingness to screen (motivated or neutral).

Word	Behavior group	No of mentions in the group	Sentiment by participants in group, mean (SD; range)	Example quotes (random 3)
Cure	BC-aware^a^ and motivated	74	0.803 (0.238; 0.177 to 1.650)	Prevention is better than cure; early detection will mean higher chance of survival; prevention is better than cure; prevention better than cure
Examination	BC-unaware and neutral	3	0.013 (0.022; 0.000 to 0.038)	If I do self-examination regularly it’s not necessary; I will do self-breast examination to look out for any early signs; prefer to do self-examination for now
Necessary	BC-unaware and neutral	3	−0.031 (0.055; −0.094 to 0.000)	Still feel not necessary at this moment; depending on if it is necessary for me; if I do self-examination regularly it is not necessary
Prevent	BC-unaware and motivated	13	0.367 (0.345; −0.177 to 1.025)	it is very important need to prevent; no prevent breast cancer; prevention of breast cancer

a BC: breast cancer.

## Discussion

### Principal Findings

This study explored the use of sentiment analysis and biterm topic modeling to examine factors influencing participants’ posteducation willingness to attend regular BC screening in a pilot study, using a specific question in a structured questionnaire with free-text questions to obtain participants’ responses. Results from the free-text analysis indicate that most participants were BC aware and had positive sentiments toward regular screening. Major topics discovered were related to the benefits of early detection and health awareness in participants who were motivated to attend BC screening. In neutral participants, mammography experience, in particular, pain during the mammogram, was the key topic highlighted.

The focus of this study was to examine the public’s views, sentiments, and concerns regarding their willingness to participate in regular BC screening following a BC education session. NLP of free-text responses provided an efficient method to filter the concerns that are prioritized by participants through biterm topic models and word count tabulations and allowed us to explore reasons by accessing the full entry. Key topics of interest to the participants may not surface in structured questionnaires, and the options are restricted by the researchers’ choice of questions. In our case, the perceived importance of screening and perceived screening barriers such as inconvenience, pain, embarrassment, and cost were assessed using a limited range of available response options (5-point Likert scale or yes or no).

A review by Hisan et al [31] categorized the use of NLP in health care into clinical applications, such as clinical documentation, medical coding, clinical decision support, and patient engagement, and public health applications, including sentiment analysis, clinical trials, and disease surveillance, demonstrating how NLP enhances health care communication and outcomes. Thackeray et al [32] analyzed over 1.3 million tweets during Breast Cancer Awareness Month in 2012, revealing that most tweets were original posts by individuals, with organizations and celebrities focusing on fundraisers and early detection, while highlighting the need for strategic, ongoing social media engagement to promote preventive behaviors and maximize outreach.

Another study analyzing tweets by Nastasi et al [33] found that nonhealth care users frequently shared unsupported claims and expressed confusion about screening guidelines, highlighting the need for better public education on BC prevention and accurate information dissemination. These studies have demonstrated the immense potential of NLP in identifying current health care barriers and equipping governmental and health care institutions with information to deliver more targeted programs. Applying NLP to this study, we found that more motivated participants were more concerned with screening utility and understood the value of regular screening, while topics brought up by neutral participants were focused on the cost (financial, mental, and time) of screening.

The participation rate for routine mammography screening is poor in Singapore’s population (<40%), a country that may be considered to be BC-aware (National Population Health Survey). In this study, we found 79% (n=3287) of our participants to be BC-aware. After completing the BC education questions, 94% (n=3908) reported being more motivated to attend screening. This is in contrast with only 63% (n=2628) who reported regular screening before the education session. BC awareness remained a significant factor in participants’ willingness to attend screening after adjusting for current screening frequency. Biterm topic modeling highlighted that the reinforcement of information on the benefits of early detection motivated the idea of attending screening.

In the area of barriers to screening, the main concern was pain among participants who were still not encouraged to screen. This echoes earlier findings that perceived pain or the experience of pain during mammography was a deterring factor for screening [34-37]. Although pain is a recognized barrier to mammography, our findings demonstrate that it remains a persistent concern. Future research should explore practical strategies to reduce discomfort and support more positive screening experiences. In contrast, other known barriers for screening, such as fatalism and cost, did not surface as concerns among our participants [4]. This may be due to multiple factors. Prior research studies on fatalism in BC were inconsistent with regard to ethnicity. While Straughan and Seow [38] found fatalism to be associated with mammography uptake in a Chinese-majority cross-sectional study, Goh et al [9] found fatalism not to be associated with mammography uptake among the Malay community [39]. In addition, some studies included older populations (aged >60 years in 2016) who were not included in BREATHE (aged 35-59 years) [40]. Furthermore, the higher income found in our population, as compared with previous research that included a larger portion of lower-income participants, may have reduced concerns about cost [37,41].

Contextual differences of the same word emphasize the need for subgroup analysis using multiple methods (examining words and sentences) to capture the information provided by free text. Notably, the word “health” was common across motivated and neutral groups. However, the context in which “health” was used differed. Motivated participants used the term to emphasize the importance of screening for maintaining one’s health. This is similar to other studies demonstrating that innate health consciousness and perceived screening importance served as facilitators to screening attendance [42-44]. In contrast, neutral participants used “health” to highlight potential health risks associated with screening and their fatalistic attitudes toward the disease.

In our BC-unaware and neutral group, the words “examination” and “necessary” were used in combination with “regular,” reflecting their perception that regular self-examination is sufficient to inform them of their health status and thus that mammography is unnecessary. This presents a gap in the understanding of mammography screening. A randomized controlled trial in Shanghai found that while breast self-examination accompanied by intensive instruction can help detect smaller fibroadenomas, it was not effective in reducing BC mortality [45]. On the other hand, Duffy et al [46] showed that mammography screening can lead to a 41% reduction in BC mortality over 10 years, along with a 25% decrease in the incidence of advanced BCs. Importantly, mammography can detect nonpalpable breast tumors, which would otherwise be missed by self-examination or clinical examination [47]. There is a need to inform the public that although regular breast self-examination is an important habit, it is not a substitute for mammography screening.

### Comparison With Previous Studies

Our previous studies in Singapore, including structured surveys, focus group discussions, in-depth interviews, and discrete choice experiments, identified cost and perceived risk (ie, women’s belief that they are at low risk of developing BC) as major determinants of participation in both standard and risk-based screening programs [11,12,42,48-50]. In contrast, the current NLP analysis revealed that cost did not emerge as a prominent factor, and perceived risk was not directly represented as a key factor, appearing instead through related concepts such as perceived screening importance. We believe that a strength of the data-driven NLP analytical approach is that it can uncover subtle insights from unstructured text that traditional survey or qualitative methods may overlook.

Considering our findings in light of the low mammography screening uptake rate in Singapore, addressing knowledge gaps and negative sentiments toward screening may increase women’s motivation to participate. Evidence suggests that individualized discussions that acknowledge women’s specific concerns can increase informed screening uptake, whereas the absence of such discussions or a lack of health care provider recommendation may discourage participation [51]. A personalized risk-based screening approach could provide a practical pathway to improve participation, as it allows for tailored communication and targeted support based on each woman’s risk profile. Our experience with the BREATHE implementation study showed that many women underestimated their BC risk, and that the perceived risk of BC can change after receiving results from a personalized predictive risk assessment [52]. However, confidence in the predicted risk result is lower among those who underestimated their risk [52]. Nonetheless, implementing such personalized approaches at scale poses challenges, including resource constraints and the need for trained personnel to deliver individualized feedback.

### Challenges of Using Online Education Tools

There were challenges in using a self-administered online questionnaire as an educational tool for BC screening. Participants appeared to be confused about the concept of early detection and cancer prevention, despite the education session emphasizing that mammography detects cancer early but does not prevent its occurrence. This was reflected in the participants’ continued use of the terms “prevention” and “prevent” when referring to mammography screening (eg, “prevention of cancer” and “prevention is better than cure”), suggesting persistent misconceptions about the purpose of screening. However, even when the conceptual distinction between cancer prevention and early detection is clarified, broader barriers (eg, health literacy, digital access, emotional concerns, and health care system influences) may continue to influence screening intentions.

### Limitations

We acknowledge several limitations of this study. There remains a gap in the validity and suitability of the tools for NLP [53]. In this study, we did not explore the accuracy of each tool in this specific use case. Compared to other studies exploring BC sentiments on social media, the availability of specific questions in this study shapes participants’ responses and provides more relevant details.

However, participants could decide whether they wanted to provide answers, and most did not, resulting in a small dataset for analysis. Additionally, answers provided by participants were short, often 1-2 sentences, plausibly due to the lack of question prompts. Future studies can include more questions with improved prompts to increase the robustness of the topics covered and obtain deeper responses from participants.

We acknowledge that domain-specific sentiment lexicons may provide added nuance for clinically focused text. However, given the lay language and short responses in our dataset, the use of a general-purpose sentiment lexicon was appropriate.

This study only included the opinions of English-speaking participants and, thus, may not capture the diverse perspectives of all women. The cohort, drawn from a risk-based screening study, may also not fully represent the general population of screening-eligible women.

Our findings are based on participants’ self-reported willingness to screen following an educational session and may not reflect actual screening behavior. The BC education session likely influenced participants’ responses, particularly in shaping positive attitudes and highlighting certain themes such as early detection. As such, the topics and sentiments derived from the free-text responses may reflect immediate posteducation reactions rather than pre-existing beliefs, representing a potential source of bias. Future studies could incorporate baseline measurements or control groups to disentangle the effects of educational interventions on sentiment and topic expression.

### Conclusion

We integrated quantitative surveys with NLP to reveal emotional dimensions of screening attitudes. Unlike existing studies focused on knowledge or intent, we showed that identical screening concepts are emotionally framed differently by motivated vs neutral participants. In practice, these findings support the need for emotionally tailored BC education and prevention strategies.

## Supplementary material

10.2196/78439 Multimedia Appendix 1 BREATHE online structured first visit questionnaire, which includes factors linked to breast cancer and related conditions, such as demographic, lifestyle, and reproductive characteristics, prior treatments, and other environmental exposures.

10.2196/78439 Multimedia Appendix 2 BREATHE education questionnaire.

10.2196/78439 Multimedia Appendix 3 Flowchart of participant selection from individuals enrolled in BREATHE.

10.2196/78439 Multimedia Appendix 4 Detailed reference classifications for sociodemographic variables (housing and educational attainment; Tables S1) and full natural language processing (NLP) pipeline documentation, including spelling corrections (Table S2), stemming rules (Table S3), stop word exclusions (Table S4), and manually adjudicated sentiment ratings (Table S5), alongside complete logistic regression models examining patient-level predictors of post-education screening willingness (Table S6), attrition analyses comparing English-language respondents to nonrespondents (Table S7) and free-text contributors to noncontributors (Table S8), exhaustive biterm topic model outputs across candidate topic solutions (k = 1–5) stratified by motivational response (more motivated vs. neutral; Tables S9–S10) and education session performance (>80% vs. ≤80%; Tables S11–S12), and a contextualized concordance analysis of high-frequency sentiment terms disaggregated by breast cancer awareness status and screening motivation (Table S13).

## Abbreviations

BC: breast cancer
BREATHE: Breast Screening Tailored for Her
NLP: natural language processing
OR: odds ratio
