# Supplementary material for: Breast Cancer Screening Knowledge and Sentiments in Singaporean Women: Mixed Methods Study Using Topic Modeling, Sentiment Analysis, and Structured Questionnaire Data
Source: J Med Internet Res. 2026 Mar 10;28:e78439. doi: 10.2196/78439 (PMC12974998; doi:10.2196/78439)
Supplement: Multimedia Appendix 1 [file jmir-v28-e78439-s001.pdf]

QUESTIONNAIRE FOR BREAST SCREENING TAILORED FOR HER

Serial No.: ☐-☐☐☐☐-☐☐

PERSONAL PARTICULARS / JOB HISTORY

Ethnic group and birth place	
1.	What is your Ethnic group?<br>☐ Chinese ☐ Malay ☐ Indian ☐ Other Specify:__________)
2.	What is your Father's Ethnic group?<br>☐ Chinese ☐ Malay ☐ Indian ☐ Other (Specify: __________)
3.	What is your Mother's Ethnic group?<br>☐ Chinese ☐ Malay ☐ Indian ☐ Other (Specify: __________)
4.	Where were you born?<br>☐ Singapore ☐ Malaysia ☐ Hong Kong/Taiwan<br>☐ PR China (Province: ____________________) ☐ Other (Specify: __________)
5.	If not born in Singapore, at what age did you come to live in Singapore?<br>(__________) years old, ☐ Don't know

**Siblings:**<br>Exclude adopted and half siblings, if don't know enter 9999: comments if any		
6.	Amongst your siblings, what order number are you?<br>(1 being the oldest)	________/C:__________________
7.	How many brothers do you have?	________/C:__________________
8.	How many sisters do you have?	________/C:__________________

Marital status	
9.	Are you….?<br>☐ never married ☐ widowed<br>☐ currently married ☐ separated or divorced

CONFIDENTIAL

QUESTIONNAIRE FOR BREAST SCREENING TAILORED FOR HER

Serial No.: ☐-☐☐☐☐-☐☐

Housing

10. Which type of housing do you live in?

- ☐ HDB or other Govt 1-3 room flat (including shop-house)
- ☐ HDB 4-room flat
- ☐ HDB 5-room flat
- ☐ HDB Executive
- ☐ Private/HUDC Apt or Condominium
- ☐ Terrace/Semi-detached/Bungalow
- ☐ Other (Specify: ....)

Income

11. What is your annual income? (See government cut off for subsidy)

- ☐ Less than $30,000
- ☐ $30,001 to $72,000
- ☐ $72,001 to $120,000
- ☐ $120,001 to $175,000
- ☐ More than $175,000
- ☐ Do not wish to disclose

CONFIDENTIAL

QUESTIONNAIRE FOR BREAST SCREENING TAILORED FOR HER

Serial No.: ☐-☐☐☐☐-☐☐

Weight and Height		(enter as 666 if unfit)
12.	How tall are you?	☐☐☐ cm
13.	How much do you weigh?	☐☐☐.☐ kg

14. Which picture best describes the way you looked at age 7 years, age 18 years and in the last 1 year.

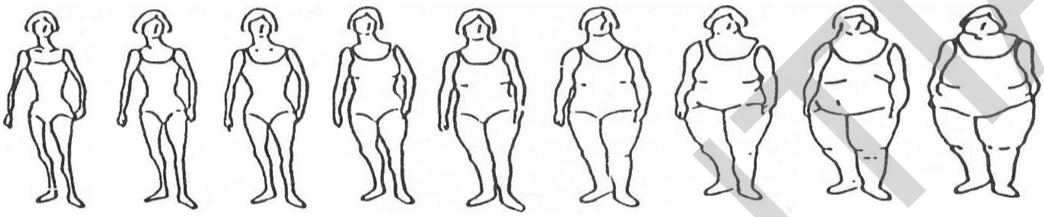

	1	2	3	4	5	6	7	8	9	Cannot Recall
**7 years**	○	○	○	○	○	○	○	○	○	○
**18 years**	○	○	○	○	○	○	○	○	○	○
**Last 1 Year**	○	○	○	○	○	○	○	○	○	○

Others

15. What is your highest academic achievement?

....

16. What is your profession? Be as specific as possible, "ex-store clerk in book shop"

....

17. Over the last 12 months were you employed?

☐ Yes

☐ No, previously employed/retired

☐ No, never employed (e.g. homemaker/students)

CONFIDENTIAL

QUESTIONNAIRE FOR BREAST SCREENING TAILORED FOR HER

Serial No.: ☐-☐☐☐☐-☐☐

LIFESTYLE & QUALITY OF LIFE

Physical Activities		
**Vigorous Physical Activities**	**Moderate Physical Activities**	**Mild Physical Activities**
Activities that take hard physical effort and make you breathe much harder than normal.	Activities that take moderate physical effort and make you breathe somewhat harder than normal.	Activities that take slight physical effort and make you breathe normally.
Examples:<br>• Heavy lifting<br>• Aerobics<br>• Vigorous cycling<br>• Vigorous swimming<br>• Running<br>• Badminton<br>• Jumping rope<br>• Hiking uphill	Examples:<br>• Carrying light loads<br>• Easy cycling<br>• Easy swimming<br>• Yoga<br>• Pilates<br>• Taiqi<br>• Qigong<br>• Jogging<br><br>Do not include brisk walking	Examples:<br>• Housework<br>• Gardening<br>• Golf<br><br>Do not include walking

Think about all the **vigorous** activities that you did in the **last 7 days**. **Vigorous** physical activities refer to activities that take hard physical effort and make you breathe much harder than normal. Think only about those physical activities that you did for at least 10 minutes at a time.

18a.	During the **last 7 days**, on how many days did you do **vigorous** physical activities like heavy lifting, aerobics, or fast bicycling?<br><br>_____ **days per week**<br><br>☐ No vigorous physical activities (Proceed to question 19a)	18b.	How much time did you usually spend doing **vigorous** physical activities on one of those days?<br><br>_____ **hours per day**<br>_____ **minutes per day**<br><br>☐ Don't know / Not sure

CONFIDENTIAL

Serial No.: ☐-☐☐☐☐-☐☐

Think about all the **moderate** activities that you did in the **last 7 days**. **Moderate** activities refer to activities that take moderate physical effort and make you breathe somewhat harder than normal. Think only about those physical activities that you did for at least 10 minutes at a time.			
19a.	During the **last 7 days**, on how many days did you do **moderate** physical activities like carrying light loads, yoga or pilates?<br>_____ **days per week**<br>☐ No moderate physical activities (Proceed to question 20a)	19b.	How much time did you usually spend doing **moderate** physical activities on one of those days?<br>_____ **hours per day**<br>_____ **minutes per day**<br>☐ Don't know / Not sure
Think about all the **mild** activities that you did in the **last 7 days**. **Mild** activities refer to activities that take slight physical effort and make you breathe normally. Think only about those physical activities that you did for at least 10 minutes at a time.			
20a.	During the **last 7 days**, on how many days did you do **mild** physical activities like housework, gardening, or golf?<br>_____ **days per week**<br>☐ No mild physical activities (Proceed to question 21a)	20b.	How much time did you usually spend doing **mild** physical activities on one of those days?<br>_____ **hours per day**<br>_____ **minutes per day**<br>☐ Don't know / Not sure
Think about the time you spent **walking** in the **last 7 days**. This includes at work and at home, walking to travel from place to place, and any other walking that you have done solely for recreation, sport, exercise, or leisure.			
21a.	During the **last 7 days**, on how many days did you **walk** for at least 10 minutes at a time?<br>_____ **days per week**<br>☐ No walking (Proceed to question 22)	21b.	How much time did you usually spend **walking** on one of those days?<br>_____ **hours per day**<br>_____ **minutes per day**<br>☐ Don't know / Not sure

CONFIDENTIAL

Think about the time you spent **sitting** on weekdays during the **last 7 days**. Include time spent at work, at home, while doing course work and during leisure time. This may include time spent sitting at a desk, visiting friends, reading, or sitting or lying down to watch television.	
22.	During the **last 7 days**, how much time did you spend sitting on **a weekday**?<br>_____ **hours per day**<br>_____ **minutes per day**<br>☐ Don't know/Not sure
23.	Did the COVID-19 situation affect your exercise routine?<br>☐ Yes, it decreased<br>☐ Yes, it increased<br>☐ No change

**Diet**	
24.	Are you currently on a special diet and have been on it in the **last 6 months**?<br>☐ Yes<br>☐ No (Proceed to question 26)
25.	State the type of diet you are on.<br>☐ Halal<br>☐ Vegan<br>☐ Vegetarian<br>☐ Keto<br>☐ Gluten-free<br>☐ Other (Specify:_____________________________)

CONFIDENTIAL

Serial No.: ☐-☐☐☐☐-☐☐

26.	Do you consume any of the following regularly in the **last 6 months**?
a.	Vitamins and health supplements<br>E.g., vitamin C supplements, multivitamins, fish oil<br>☐ Yes<br>☐ No<br>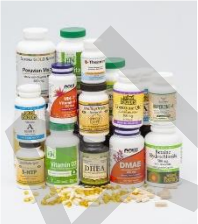
b.	Traditional or herbal supplements<br>E.g., Traditional Chinese Medicine (TCM), Jamu, Ayurveda, essence of Chicken, bird's nest<br>☐ Yes<br>☐ No<br>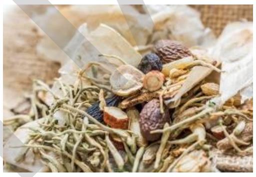
c.	Probiotics<br>E.g., Yakult, Vitagen, kefir, fermented food, yogurt, Kombucha<br>☐ Yes<br>☐ No<br>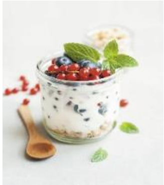
27a.	Tick the box which most accurately reflects how much (on average) you like **durian** (not necessarily how much you actually consume). If you don't know, or don't remember ever having tried or encountered, please select "Not applicable".<br>☐ Dislike a lot<br>☐ Dislike a little<br>☐ Neither like nor dislike (neutral) (Proceed to question 28)<br>☐ Like a little<br>☐ Like a lot<br>☐ Not applicable (Proceed to question 28)
b.	I like or dislike **durian** because of the:<br>☐ Smell<br>☐ Taste<br>☐ Others, please specify:___________________

CONFIDENTIAL

Serial No.: ☐-☐☐☐☐-☐☐

Smoking

28. Have you smoked on a regular basis or more than a total of 100 cigarettes?

☐ Yes

☐ No (Proceed to question 30)

29. How much did you smoke on average during different periods of your life? Check the box for each time period

Age	Number of cigarettes/day					
	NA	0	1-5	6-10	11-20	>20
Before age 20 years	☐	☐	☐	☐	☐	☐
20-29 years	☐	☐	☐	☐	☐	☐
30-39 years	☐	☐	☐	☐	☐	☐
40-49 years	☐	☐	☐	☐	☐	☐
50-59 years	☐	☐	☐	☐	☐	☐

Alcohol

30. Have you ever drunk alcohol (such as beer, rice wine, red/white wine or spirit/hard liquor) more than once a month, on average?

☐ Yes

☐ No (Proceed to question 35)

31. How old were you when you start drinking one or more times per month?

(..............................) years old ☐ Don't recall

A STANDARD alcoholic drink is defined as a can (330 ml) of regular beer, half a glass (100 ml) of wine or 1 nip (30 ml) of spirit. (Ministry of Health).

32. During the past year, how often did you usually have any kind of drink containing alcohol? (Choose only one)

☐ Every day

☐ ____ time(s) a week (indicate a number between 1 and 6)

☐ ____ time(s) a month (indicate any number)

☐ ____ time(s) a year (indicate any number)

☐ Don't recall

CONFIDENTIAL

33.	During the past year, how many alcoholic drinks did you have on a typical day when you drank alcohol?<br>☐ 1 drink<br>☐ 2 drinks<br>☐ 3 drinks<br>☐ Others, please specify:___________<br>☐ Don't recall
34.	During the past year, how often did you have 4 or more drinks containing any kind of alcohol within a two-hour period? Choose only one:<br>☐ Every day<br>☐ ____ time(s) a week (indicate a number between 1 and 6)<br>☐ ____ time(s) a month (indicate any number)<br>☐ ____ time(s) a year (indicate any number)<br>☐ Don't recall

**Quality of Life**	
35.	How would you rate your overall <u>health</u> during the past week?<br>☐ Very poor ☐ Poor ☐ Fairly poor ☐ Average ☐ Fairly good ☐ Good ☐ Excellent
36.	How would you rate your overall <u>quality of life</u> during the past week?<br>☐ Very poor ☐ Poor ☐ Fairly poor ☐ Average ☐ Fairly good ☐ Good ☐ Excellent

CONFIDENTIAL

Serial No.: ☐-☐☐☐☐-☐☐

MEDICAL HISTORY

37.	Have you ever been diagnosed with any of the following diagnosis?			
	**State year when first diagnosed / Enter (Year)* as 9999 if not known**	**No**	**Yes**	(***Year***)*
	Heart attack / IHD	☐	☐	☐☐☐☐
	Hypertension/ High blood pressure	☐	☐	☐☐☐☐
	Hyperlipidemia/ High cholestrol	☐	☐	☐☐☐☐
	Asthma	☐	☐	☐☐☐☐
	Renal Disease	☐	☐	☐☐☐☐
	Stroke	☐	☐	☐☐☐☐
	Depression	☐	☐	☐☐☐☐
	Breast related diseases: (Specify:................................)	☐	☐	☐☐☐☐
	Others: (Specify:..)	☐	☐	☐☐☐☐
	Others: (Specify:..)	☐	☐	☐☐☐☐
38.	Do you have diabetes? Which year were you diagnosed?<br>☐ Yes<br>☐ No (Proceed to question 40)			(***Year***)*<br>☐☐☐☐
39.	If Yes, are you currently taking any medication (tablets or insulin) for your diabetes?<br>☐ Yes, tablets ☐ Yes, insulin ☐ Yes, both insulin and tablets<br>☐ No			
40.	Have you ever previously been diagnosed with a cancer?<br>☐ Yes<br>☐ No or ☐ Don't know			

RISK FACTORS

**Benign Lump/ Cyst**	
41.	Did you ever have biopsy for a benign lump or cyst in the breast?<br>☐ Yes<br>☐ No or ☐ Don't recall (Proceed to question 43)

CONFIDENTIAL

Serial No.: ☐-☐☐☐☐-☐☐

42.	If yes, how many times of biopsy had you gone? ________
43.	Did you ever have surgery for a benign lump or cyst in the breast?<br>☐ Yes ☐ No ☐ Don't recall
44.	Did you ever have gynaecological surgery?<br>☐ Yes<br>☐ No or ☐ Don't recall (Proceed to question 46)
45.	If Yes, please state type of surgery and what year: Enter not known as 9999
	☐ Sterilisation ☐☐☐☐<br>☐ Removal of one ovary ☐☐☐☐<br>☐ Removal of both ovaries ☐☐☐☐<br>☐ Removal of uterus ☐☐☐☐<br>☐ Removal of part of cervix (conisation) ☐☐☐☐<br>☐ Other surgery: .... ☐☐☐☐

**Family History of Breast Cancer**	
46.	Have any of your family and/or relatives been diagnosed with breast cancer?<br>☐ Yes<br>☐ No or ☐ Don't know (Proceed to question 48)
47.	If Yes, who and at what age - (enter **999,** if not known)
	☐ .... at age of - ☐☐☐ years old; ☐ Estimated<br>☐ .... at age of - ☐☐☐ years old; ☐ Estimated<br>☐ .... at age of - ☐☐☐ years old; ☐ Estimated<br>☐ .... at age of - ☐☐☐ years old; ☐ Estimated

CONFIDENTIAL

Serial No.: ☐-☐☐☐☐-☐☐

Family History of Ovarian Cancer	
48.	Have your mother/sister/daughter and/or any other relatives been diagnosed with ovarian cancer?<br>☐ Yes<br>☐ No or ☐ Don't know (Proceed to question 50)
49.	If Yes, who and at what age - (enter **999,** if not known)
	☐ .... at age of - ☐☐☐ years old; ☐ Estimated<br>☐ .... at age of - ☐☐☐ years old; ☐ Estimated<br>☐ .... at age of - ☐☐☐ years old; ☐ Estimated<br>☐ .... at age of - ☐☐☐ years old; ☐ Estimated

Family History of Other Cancers	
50.	Have any of your family and/or relatives been diagnosed with any other cancer?<br>☐ Yes<br>☐ No or ☐ Don't know (Proceed to question 52)
51.	If Yes, who, what cancer and at what age (enter **999,** if not known)
	☐ .... at age of - ☐☐☐ years old; ☐ Estimated<br>☐ .... at age of - ☐☐☐ years old; ☐ Estimated<br>☐ .... at age of - ☐☐☐ years old; ☐ Estimated<br>☐ .... at age of - ☐☐☐ years old; ☐ Estimated

MENSTRUATION, PREGNANCY AND CHILDBIRTH

Menstruation	
52.	How old were you at your first menstruation?<br>☐☐ years old ☐ Don't recall

CONFIDENTIAL

Pregnancy

53. Have you ever been pregnant?

☐ Yes ☐ No (Proceed to question 56)

54. How many children do you have?

....

55. Please state birth year and birth weight for all children you gave birth to and number of months you breast fed each child.

	Child's Birthyear Enter not known as 9999	Birthweight gram	Don't recall	Breastfeeding Months	Don't recall
1	☐☐☐☐	☐☐☐☐	☐	☐☐	☐
2	☐☐☐☐	☐☐☐☐	☐	☐☐	☐
3	☐☐☐☐	☐☐☐☐	☐	☐☐	☐
4	☐☐☐☐	☐☐☐☐	☐	☐☐	☐
5	☐☐☐☐	☐☐☐☐	☐	☐☐	☐
6	☐☐☐☐	☐☐☐☐	☐	☐☐	☐
7	☐☐☐☐	☐☐☐☐	☐	☐☐	☐
8	☐☐☐☐	☐☐☐☐	☐	☐☐	☐
9	☐☐☐☐	☐☐☐☐	☐	☐☐	☐
10	☐☐☐☐	☐☐☐☐	☐	☐☐	☐
11	☐☐☐☐	☐☐☐☐	☐	☐☐	☐
12	☐☐☐☐	☐☐☐☐	☐	☐☐	☐

CONFIDENTIAL

Conception Treatment		
56.	Have you ever been treated for infertility?<br>☐ Yes<br>☐ No (Proceed to question 58)	
57.	If Yes, what treatments did you receive and how old were you?	Enter not known as 999
	☐ Surgery	☐☐☐ years old
	☐ *In vitro* fertilisation	☐☐☐ years old
	☐ *In vitro* fertilisation	☐☐☐ years old
	☐ Hormone stimulation (for ovulation)	☐☐☐ years old
	☐ Hormone stimulation (for ovulation)	☐☐☐ years old
	☐ Others:	☐☐☐ years old
	☐ Don't recall	
58.	Have you ever used oral contraceptives, including injections for birth control?<br>☐ Yes<br>☐ No (Proceed to question 61)	
59.	If yes, when did you start treatment?<br>Year ☐☐☐☐ Month ☐☐<br>☐ Don't recall	
60.	Are you still using oral contraceptives?<br>☐ Yes<br>☐ No, stopped in ☐☐☐☐ (Year) ☐ Don't recall	

CONFIDENTIAL

CONFIDENTIAL

Menopause

61.	Are you still menstruating?<br>☐ Yes, I still have 'natural' menstruation bleedings. (Proceed to question 63)<br>Last bleeding ☐☐☐☐ - ☐☐ Year, Month ☐ Don't recall<br>☐ Yes, I have bleeding because of ongoing hormone treatment<br>prior to hormone treatment: (Proceed to question 63)<br>Last bleeding ☐☐☐☐ - ☐☐ Year, Month ☐ Don't recall<br>☐ No

62.	If No, please state reason and what year menstruation stopped:	Enter not known as 9999
	☐ It stopped by itself	☐☐☐☐
	☐ Uterus was removed	☐☐☐☐
	☐ Ovaries were removed	☐☐☐☐
	☐ Hormone treatment was stopped	☐☐☐☐
	☐ Other reason:	☐☐☐☐
	☐ Don't recall	

Hormone Replacement Treatment

63.	Have you ever used hormone medication?<br>☐ Yes<br>☐ No or ☐ Don't recall (Proceed to question 66)
64.	At what year did you start treatment?<br>Year ☐☐☐☐ Type of treatment: ________________<br>☐ Don't recall
65.	Are you still taking hormone medication?<br>☐ Yes<br>☐ No, stopped in ☐☐☐☐ (Year) ☐ Don't recall

QUESTIONNAIRE FOR BREAST SCREENING TAILORED FOR HER

Serial No.: ☐-☐☐☐☐-☐☐

BREAST EXAMINATION & SCREENING

Breast Self-Examination

Interviewer to read out to study participant:

"A breast self-exam is a method used in an attempt to detect early breast cancer . This is what is looks like when you do a breast self-exam." *(show picture)*

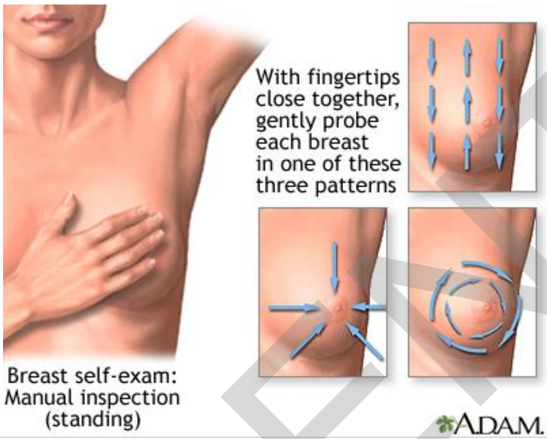

66.	Have you heard of a breast self-examination?<br>☐ Yes<br>☐ No (Proceed to question 71)
67.	Have you ever done a breast self-examination?<br>☐ Yes, last breast self-examination in ☐☐☐☐<br>☐ No (Proceed to question 70)
68.	How often have you done breast self-examination in the past 6 months?<br>☐ Regularly, once a month or more<br>☐ Whenever I remember or am reminded of it<br>☐ Others, please specify: ________________
69.	Why do you do breast self-examination? (Proceed to question 71)<br>[DO NOT READ THIS LIST OUT]<br>☐ Know the importance of screening<br>☐ Have a current/previous gynecological problem<br>☐ Advised by doctors/nurses<br>☐ My family/friends/colleagues encouraged me<br>☐ Read/heard about it/saw an advertisement about breast self-examination<br>☐ Others (please specify: ____)

CONFIDENTIAL

70.	Can you tell me why you had not done a breast self-examination?<br>[DO NOT READ THIS LIST OUT]<br>Please tick all that apply as indicated by study participant<br>☐ Not necessary as I am healthy<br>☐ Not at risk<br>☐ Not useful<br>☐ Too old<br>☐ Too young<br>☐ Afraid of possible side effects<br>☐ Afraid of knowing the results<br>☐ Inconvenient<br>☐ Not important<br>☐ No time due to work or family commitments<br>☐ Cannot do anything if breast cancer is detected<br>☐ Didn't know what to do<br>☐ Painful test<br>☐ Embarrassing<br>☐ Not suggested by my doctors or nurses<br>☐ Never thought about it before<br>☐ Others (please specify: ______________________________)

**Mammogram Screening**	
71.	Have you ever heard of mammogram before this study?<br>☐ Yes<br>☐ No (Proceed to question 75)
72.	Have you ever had a mammogram done?<br>☐ Yes, last mammography exam in ☐☐-☐☐☐☐ (Month, Year: [YYYY])<br>Comment (e.g. location): ....<br>☐ No (Proceed to question 74)

CONFIDENTIAL

73.	Why did you go for the mammogram? (Proceed to question 75)<br>Please tick all that apply as indicated by study participant [DO NOT READ THIS LIST OUT]<br>☐ Know the importance of screening<br>☐ Have a current/previous gynecological problem<br>☐ Advised by doctors/nurses<br>☐ My family/friends/colleagues encouraged me<br>☐ Read/heard about it/saw an advertisement about mammogram<br>☐ Received a letter to encourage me to go for screening<br>☐ Ad-hoc health screening / Routine check-up<br>☐ Others (please specify: ....)
74.	Why had you not gone for a mammogram?<br>[DO NOT READ THIS LIST OUT]<br>Please tick all that apply as indicated by study participant<br>☐ Not necessary as I am healthy<br>☐ Not at risk<br>☐ Too old<br>☐ Too young<br>☐ Cost of the test is too expensive<br>☐ Afraid of possible side effects<br>☐ Afraid of knowing the results<br>☐ Inconvenient<br>☐ Not important<br>☐ No time due to work or family commitments<br>☐ Cannot do anything if breast cancer is detected<br>☐ Didn't know where to go<br>☐ Painful test<br>☐ Embarrassing<br>☐ Not suggested by my doctors or nurses<br>☐ Never thought about it before<br>☐ Others (please specify: ....)

CONFIDENTIAL

Ultrasound Screening	
75.	Have you ever heard of breast ultrasound screening?<br>☐ Yes<br>☐ No (Stop here)
76.	Have you ever had a breast ultrasound screening done?<br>☐ Yes, last breast ultrasound exam in ☐☐-☐☐☐☐ (Month / Year: [YYYY])<br>Comment (e.g. location): ....<br>☐ No (Proceed to question 78)
77.	Why did you go for the breast ultrasound screening? (Stop here)<br>[DO NOT READ THIS LIST OUT]<br>Please tick all that apply as indicated by study participant<br>☐ Know the importance of screening<br>☐ Have a current/previous gynecological problem<br>☐ Advised by doctors/nurses<br>☐ My family/friends/colleagues encouraged me<br>☐ Read/heard about it/saw an advertisement about ultrasound<br>☐ Ad-hoc health screening / Routine check-up<br>☐ Others (please specify: ....)

CONFIDENTIAL

78.	Why have you not gone for a breast ultrasound screening?<br>[DO NOT READ THIS LIST OUT]<br>Please tick all that apply as indicated by study participant<br>☐ Not necessary as I am healthy<br>☐ Not at risk<br>☐ Too old<br>☐ Too young<br>☐ Cost of the test is too expensive<br>☐ Afraid of possible side effects<br>☐ Afraid of knowing the results<br>☐ Inconvenient<br>☐ Not important<br>☐ No time due to work or family commitments<br>☐ Cannot do anything if breast cancer is detected<br>☐ Didn't know where to go<br>☐ Embarrassing<br>☐ Not suggested by my doctors or nurses<br>☐ Never thought about it before<br>☐ Others (please specify: ....)

CONFIDENTIAL
