# Supplementary material for: Breast Cancer Screening Knowledge and Sentiments in Singaporean Women: Mixed Methods Study Using Topic Modeling, Sentiment Analysis, and Structured Questionnaire Data
Source: J Med Internet Res. 2026 Mar 10;28:e78439. doi: 10.2196/78439 (PMC12974998; doi:10.2196/78439)
Supplement: Multimedia Appendix 3 [file jmir-v28-e78439-s003.docx]

Supplementary Figure 1. Flowchart of participant selection from individuals enrolled in BREATHE.
